# Malignant otitis externa

**DOI:** 10.1016/S1808-8694(15)31137-X

**Published:** 2015-10-20

**Authors:** Gilberto Gattaz, Lucimar Santos Sperotto, Larissa Matos Rebouças

**Affiliations:** 1 Associate Professor PUC-SP. Otorhinolaryngologist - Serviço de ORL do Hospital dos Defeitos da Face; 2 ENT Resident - Hospital dos Defeitos da Face. Intern; 3 ENT Resident - Hospital dos Defeitos da Face. Intern

**Keywords:** immunosuppressed, necrotizing otitis externa, pseudomonas aeruginosa

## INTRODUCTION

Malignant otitis externa (MOE) is a severe, invasive and necrotizing infectious disease that starts in the external auditory meatus (EAM) and may progress to the parotid region, mastoid, middle ear and skull base^1^.

It affects mainly the diabetic, the elderly and immunosuppressed patients. Main etiological agent: P. aeruginosa.

Symptoms: Otalgia, mal-odorous otorrhea and local edema

Diagnosis is carried out through anamnesis, clinical exam, finding the germ and complementary exams. Temporal bone CT Scans allows for the identification of MOE erosions and the MRI defines its expansion to the skull base. Although non-specific, ESR (erythrocyte sedimentation rate) is a parameter that measures MOE's evolution^2^,^3^.

Differential diagnosis: benign neoplasia of the EAM, malignant neoplasia of the EAM and cholesteatoma^4^.

Treatment: IV ciprofloxacin and continue with it orally until HSS normalization.

## CASE PRESENTATION

JB, 53 years old, with intense left side otalgia, irradiating to the temporal region and half of his face.

It's been developing for one month, after swimming in the ocean, treated with ear drops, without improvement. Patient reports never having ear problems or D. mellitus.

Otoscopy: EAM edema, purulent otorrhea and tenderness. Tympanic membrane was not visible. Under the assumption of an external otitis media, we medicated the patient with IM betamethasone, amoxicillin 1.5g/day per os and topical ciprofloxacin.

The patient did not show significant improvement. We then requested a CT scan and prescribed ciprofloxacin per os 1.5g/day and topical drops for maintenance treatment.

**Figure 1:**
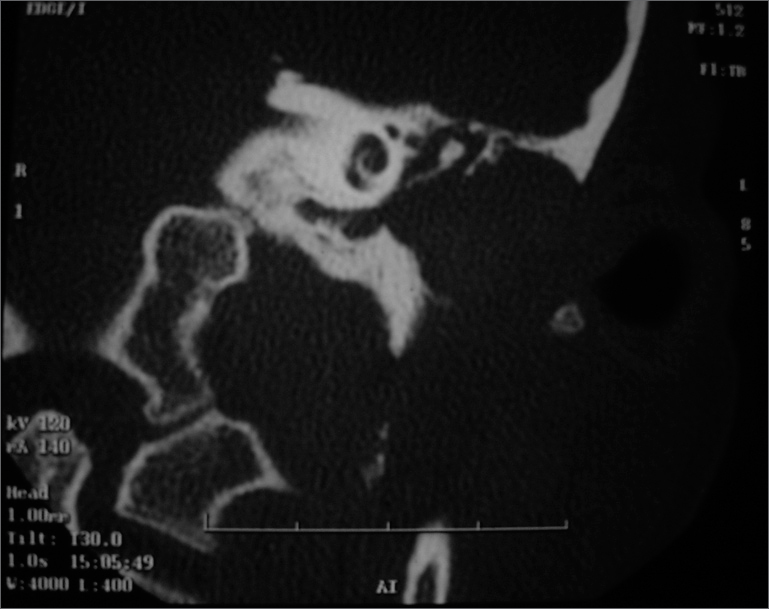
Temporal bone CT Scan - Left Ear.

CT scan (figure 1) showed a hyperdense lesion occluding all the EAM, erosion of its walls and the petrous bone, with inflammatory mastoiditis. Differential diagnosis did not rule out a neoplastic lesion; however the latter was ruled out by MRI. Glucose levels, CBC and ESR within normal ranges.

Considering a diagnosis of MOE, we chose to use 750mg of ciprofloxacin BID per os.

After one month without complaints, otoscopy showed a significant improvement.

Tonal audiometry: mixed hearing loss, more intense in the higher frequencies with a 10 to 25dBHL bone-air gap.

Tympanometry in −250 dcPa. No stapedial reflexes.

## DISCUSSION

D. Mellitus may represent a predisposing factor for MOE^1^, ^2^, ^5^, ^6^; however all tests were normal. The germ was not found because of previous treatment. MOE diagnosis was based on the anamnesis and the patient's response to treatment. We have to bear in mind that the patient had not had any prior ear infection. The inefficiency of the previous treatment allowed us to consider the possibility of MOE.

CT scan findings (Figure 1) have corroborated that information. Please notice that Chaussé's spur, epitympanum and the incus-malleus joint are intact. The mastoid disease is a reactional inflammatory process. MRI ruled out malignant neoplasia or cholesteatoma. The disease progression and the image findings indubitably led us to conclude for diagnosis of MOE in a patient lacking conventional predisposing factors.

Oral ciprofloxacin or ceftazidime and IV cefoperazone, associated to ciprofloxacin per os represent the drug treatment of choice. In the present case, we chose to use ciprofloxacin per os for 2 weeks3, with both clinical and otoscopic improvement. Considering the disease's course and the possibility of sequela and fatal complications, we stress the need to be attentive towards this condition, and not underestimate any external otitis, always suspect of MOE, especially in cases of unexpected developments regarding the prescribed treatment.
